# Supplementary material for: Predicting visual function by interpreting a neuronal wiring diagram
Source: Nature. 2024 Oct 2;634(8032):113–23. doi: 10.1038/s41586-024-07953-5 (PMC11446822; doi:10.1038/s41586-024-07953-5)
Supplement: Supplementary file 6 — CRF and ERF predictions for individual TmY4 and TmY9 cells. Analogous to Supplementary Data 3, but for TmY target types. Shown are the top four monosynaptic pathways, the strongest pathway passing through each of the top ten intermediary types (ranking from Extended Data Fig. 7), and the trisynaptic pathway Tm1–TmY–Dm3–TmY (see the section entitled Prediction of spatial normalization). [file 41586_2024_7953_MOESM6_ESM.zip › DataS4/TmY9qΓèÑ/720575940631215865.pdf]

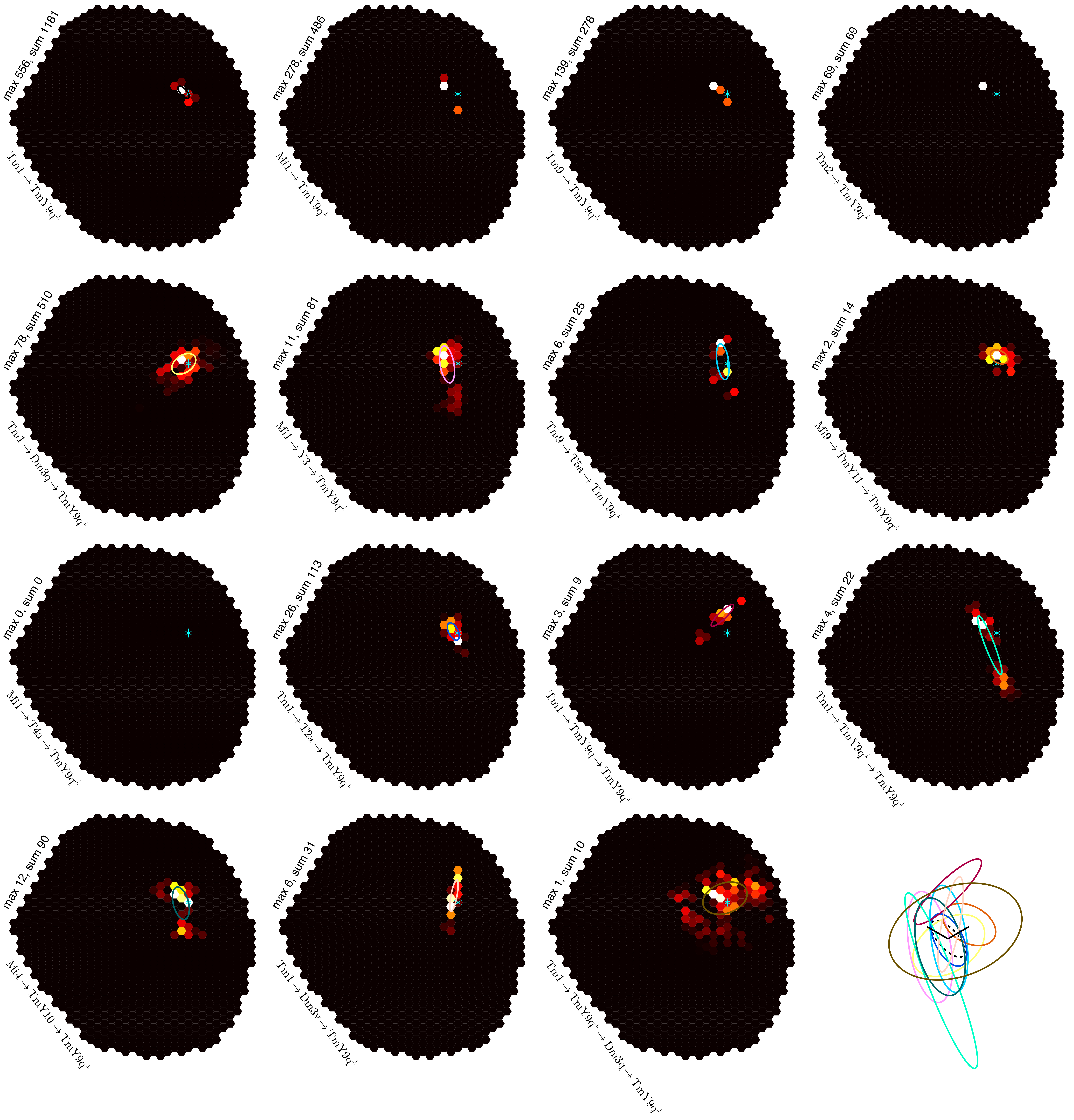

max 556, sum 1181
Tm1 → TmY9q⊥
max 278, sum 486
Mi1 → TmY9q⊥
max 139, sum 278
Tm9 → TmY9q⊥
max 69, sum 69
Tm2 → TmY9q⊥
max 78, sum 510
Tm1 → Dm3q → TmY9q⊥
max 11, sum 81
Mi1 → Y3 → TmY9q⊥
max 6, sum 25
Tm9 → T5a → TmY9q⊥
max 2, sum 14
Mi9 → TmY11 → TmY9q⊥
max 0, sum 0
Mi1 → T4a → TmY9q⊥
max 26, sum 113
Tm1 → T2a → TmY9q⊥
max 3, sum 9
Tm1 → TmY9q → TmY9q⊥
max 4, sum 22
Tm1 → TmY9q⊥ → TmY9q⊥
max 12, sum 90
Mi4 → TmY10 → TmY9q⊥
max 6, sum 31
Tm1 → Dm3v → TmY9q⊥
max 1, sum 10
Tm1 → TmY9q⊥ → Dm3q → TmY9q⊥
